# ENGAGING OLDER ADULTS VIA SOCIAL MEDIA DURING A PANDEMIC: SUCCESSFUL LEARNING EXPERIENCES FOR STUDENTS AND ADULTS

**DOI:** 10.1093/geroni/igad104.2812

**Published:** 2023-12-21

**Authors:** Krista Cox, Tracey Zeiner, Jennifer Moore, LaVona Traywick, Robin McAtee

**Affiliations:** A. T. Still University, Fort Smith, Arkansas, United States; Arkansas Colleges of Health Education School of Occupational Therapy, Fort Smith, Arkansas, United States; AR Colleges of Health Education, Fort Smith, Arkansas, United States; Arkansas Colleges of Health Education, Fort Smith, Arkansas, United States; University of Arkansas for Medical Sciences, Little Rock, Arkansas, United States

## Abstract

The Arkansas Colleges of Health Education and the Arkansas Geriatric Education Collaborative collaborated on a project to educate senior adults about the use of assistive technologies (AT) for self-care, home management, community engagement, and leisure participation to help them live safely and independently. Occupational and physical therapy students collaborated with 10 community dwelling senior adults to research, develop, and disseminate 27 video vignettes shared via Facebook. These vignettes were developed and shown during a time when senior adults were encouraged to isolate during the pandemic. As of January 2023, the outcomes of sharing the 27 video vignettes on Facebook included: a total of 17,204 views, the most viewed was “Lighting and Magnification” at 1,000+ views, followed by “Sewing” at 992 views, the least viewed was “Fishing” at 147 views. Older adult viewers feedback included: contact with ACHE to ask how to purchase the assistive devices; sharing the information gained with their family members and others; purchase of devices; providing a resource for AT assistance for OA. Additionally, the senior adults that participated in the project reported a better understanding of the AT after presenting in the videos. In conclusion, these results indicate that creating the video vignettes and sharing them on Facebook had an extensive reach beyond the 27 individual sessions. This also demonstrated that the viewers were actively engaged as noted by their questions and comments.

